# Supplementary material for: Disorder enabled band structure engineering of a topological insulator surface
Source: Nat Commun. 2017 Feb 3;8:14081. doi: 10.1038/ncomms14081 (PMC5296772; doi:10.1038/ncomms14081)
Supplement: Supplementary Information — Supplementary Figures, Supplementary Notes and Supplementary References. [file ncomms14081-s1.pdf]

SUPPLEMENTARY NOTE 1: RESONANCE STATE NUMBER, DEFECT NUMBER, AND DEFECT SPECIATION

The carrier densities of samples in the M_2X_3 material family can vary widely with growth conditions, but are commonly on the order of 1×10^{19} cm^{-3}, and have been largely attributed to point lattice defects such as chalcogen vacancies. Assuming two doped electrons per vacancy, a carrier density of 1×10^{19} cm^{-3} corresponds to a defect density of $\rho = 0.08\%$ per 2D lattice cite within an M_2X_3 TI quintuple layer, similar to the point defect density of $\rho = 0.06\%$ seen by STM in our investigations of Bi_2Se_3 [1]. Based on this correspondence, the defect species measured in Fig. 2b of the main text can be attributed as the principal electron donor of the n-type Bi_2Se_3 crystal.

A very thorough recent study on Bi_2Se_3 has found that defects near the cleaved surface in Se-poor or stoichiometric synthesis are primarily selenium vacancies located in the 3rd atomic layer from the surface [2] (center of the quintuple layer). The density of these defects can be controlled over an extremely wide range (up to $\rho = 0.35\%$ in Supplementary Figure 1). Selenium-rich synthesis results in somewhat similar point defects that are likewise charge donors, and appear to represent interstitial Se atoms [2]. Quench-cooled samples can exhibit a range of other defects, such as seemingly amphoteric sites on which bismuth replaces selenium in the lattice. The present study has focused on the defect density range from $0.02\% \leq \rho \leq 0.18\%$, as it is not well known if good long-range (micron to millimeter scale) crystallinity can be achieved for highly non-stoichiometric synthesis. It should be noted that while these defects do act as dopants, numerous studies have demonstrated methods such as bulk doping, surface dosing, and electrostatic gating [3–6] by which the chemical potential can be shifted as a separate parameter.

Supplementary Figure 2 shows ARPES spectral functions and twist velocities predicted for several different U values, which may be relevant to different species of defect. We note that a band connecting to the D_1 feature from below becomes visible for larger values of U. A momentum-resolved plot of the ratio of the twist velocity to the apparent band slope in the upper D_0 Dirac cone is also shown in Supplementary Figure 3, based on Fig. 3c of the main text ($U = -45$ eV, $\rho = 0.18\%$). This plot illustrates that both the band dispersion and the twist velocity approach zero at large momentum, as the band disperses into the seemingly gapped region beneath the D_1 feature. The twist velocity appears to decay more

rapidly than the band slope for momenta $k \gtrsim 0.05\mathring{A}$, however it is difficult to trace the band slope with sufficient accuracy in this region.

Histograms of the modeled participation ratio are compared with DOS and band structure calculations in Supplementary Figure 4, with dashed lines bracketing the electronic structure most influenced by defects. Summing all states between the dashed lines gives a number equal to twice the number of defects, showing that approximately 2 resonance states with large participation ratios can be identified per defect. This is the behavior that would be expected if the resonance states were energetically-isolated bound states, as all bound states are doubly degenerate in the absence of time reversal symmetry breaking.

SUPPLEMENTARY NOTE 2: ROTATIONAL ANISOTROPY OF DEFECTS

The STM image of a defect resonance state in Fig. 1a has 3-fold rotational symmetry, with 3 bright spots and 3 dark spots. At a fixed radius, the ratio of intensity maxima to minima is approximately $R = \frac{I_{max}}{I_{min}} \lesssim 1.5$, and systematic error in the background may add up to 0.2 to the ratio. Defining normalized basis states for for s- and f-wave components of the wavefunction as $\Psi_s(\theta) = 1/\sqrt{2\pi r}$ and $\Psi_f(\theta) = sin(6\theta)/\sqrt{\pi r}$, the ratio of maximum to minimum intensities can be calculated as $R = ((A_s + \sqrt{2} \times A_f)/(A_s - \sqrt{2} \times A_f))^2$, where A_s (A_f) is the quantum amplitude of the s-wave (f-wave) component. A ratio $R = 1.7$ is obtained with an f-wave partial density of states of ${A_f}^2 = 0.01$, giving an upper bound of roughly 1% for the f-wave admixture.

Hexagonal warping of the Fermi surface is not included in this study, as it scales with the third power of momentum [7], and the present focus is on small momenta close to the Dirac point. With realistic parameters ($\lambda \lesssim 250eV\mathring{A}^{-3}$ for $Bi_2(Se/Te)_3$ [7]) the effect of hexagonal warping is a very slight increase to the f-wave component of resonance states, to a degree that is not easily visible on the color scale in Fig. 1(a) of the main text.

Momentum-resolved spectra are highly isotropic, and have been rotationally averaged in the main text to provide a more continuous map of momentum dependence in the ARPES spectral function. When plotted along a single momentum axis, the features are effectively identical, but are broken into broadly spaced discrete momentum states that intersect the chosen momentum axis. An example of what this looks like for the ARPES simulations in Fig. 3 is shown in Supplementary Figure 5.

The energy cutoff of W=400 meV is chosen to roughly match the range over which a Dirac-like dispersion is known to be realized in $Bi_2Se_{3-x}Te_x$. This cutoff means that the basis cannot nicely resolve the atomic structure of defects. In the main text, point defects are created as 3-site clusters with a perturbation strength of U/3 on each site. However, changing this to a larger 6-site triangular cluster (U/6 on each site) or a single atomic site perturbation has no qualitative impact on simulted spectra, as seen in Supplementary Figure 6. The value of U required to maintain the resonance state energy relative to the Dirac point is slightly smaller for larger clusters, as described in the figure caption. However, setting the resonance state energy to match the experimental scenario for defects discussed in the main text results in very similar spectra, which are just past the crossover point of manifesting an intensity local maxiumum at the D_1 Dirac point.

SUPPLEMENTARY NOTE 3: ADDITIONAL MODELING DETAILS

Uniformly distributed random numbers used to generate the defect configurations were obtained using a Mersenne twister generator [8]. We note that the distribution of defects observed on real Bi_2Se_3 surfaces is also highly uniform and uncorrelated on a ~100nm scale, as seen from the Poisson fit in Supplementary Figure 1b. Full diagonalization of the Hamiltonian was performed using LAPACK drivers [9], with numerical accuracy at least 5 orders of magnitude better than the nearest significant (or visually resolvable) digit of extracted quantities. Individual simulations of system sizes of 300×300 sites or greater yielded visually smooth and reproducible ARPES spectra. Smaller systems are only considered in Fig. 4 of the main text, where configurational averaging over multiple defect distributions is used to achieve smooth trend curves for the participation ratio and twist velocity.

The simulation in Fig. 3f is seeded from defect locations observed by STM in Supplementary Figure 1a, rather than a random distribution. A 10nm border was cropped from the edge of the simulation output, to reduce inaccuracy associated with the repeating boundary conditions of the model. Unfortunately, the mapped region has a surface area more than 50% smaller than the 300×300 system size qualitatively associated with good convergence. The small system size contributes to roughness in the LDOS curves and is problematic for extracting the LDOS curve minima, which are intrinsically unstable against noise. To obtain a reliable spatially-resolved estimate of the LDOS minimum, LDOS curves were convoluted

by a 20meV peak width at half maximum (pwhm) Gaussian, and the local minima were averaged over an 8nm radius. The 16nm diameter of averaging is similar to the wavelength of electrons roughly 200meV from the Dirac point, and was chosen to maximize accuracy while remaining near the resolution limit of real physical effects near the Dirac point energy.

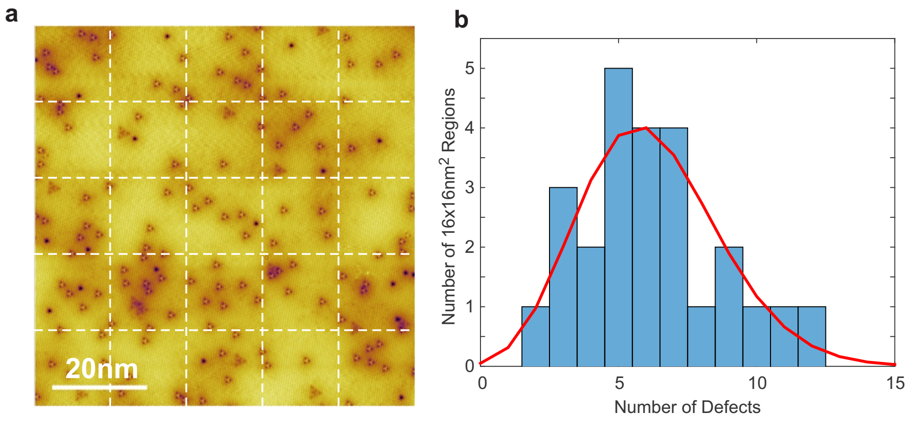

Supplementary Figure 1: **Defect distribution**: **a**, An STM topography map of the Se-poor surface investigated in Fig. 3e-f of the main text. Triangular defects in this image can be attributed as Se vacancies in the 3rd atomic layer from the surface, while a small number of smaller and darker spots are associated with Se vacancies in the top atomic layer. **b**, A histogram of the density distribution of Se vacancy defects in the partitioned 16×16 nm^2 regions of panel (a) is overlaid with the Poisson distribution. The image in panel (a) is adapted with permission from Ref. [2], copyrighted by the American Physical Society.

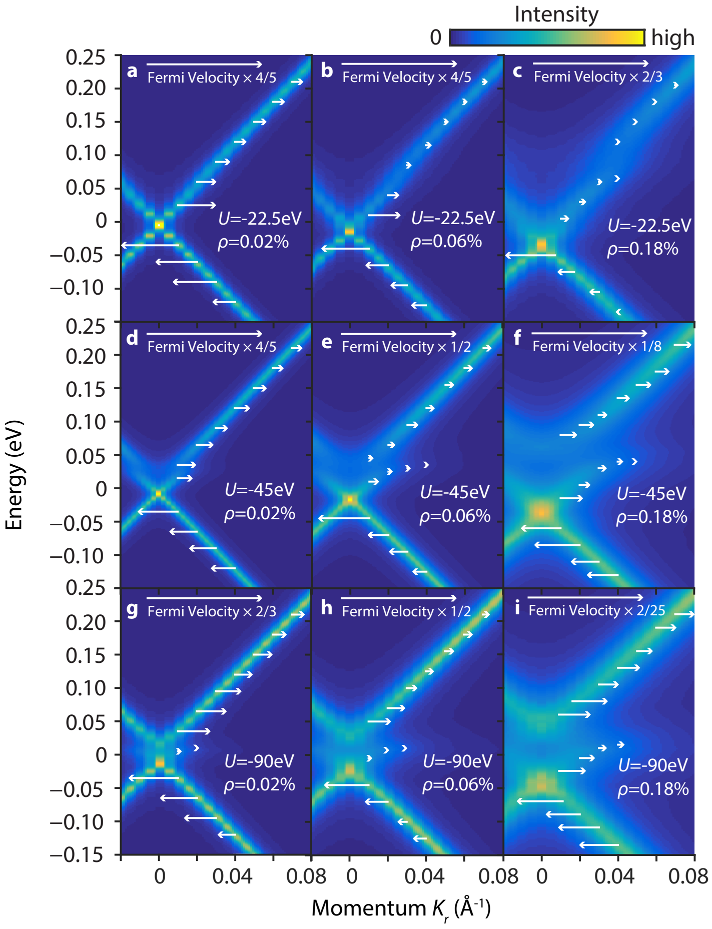

Supplementary Figure 2: **Emergent structure for different defect potentials**: **a-c**, The momentum resolved spectral function of a large TI surface with randomly distributed $U = -22.5$ eV point defects is shown as a function of defect density 'ρ'. Arrows indicate radial axis twist velocity $v_\theta(E, \mathbf{K})$. Panels (d-f) and (g-i) show the density dependence for defect potentials of $U = -45$ and $U = -90$ eV, respectively.

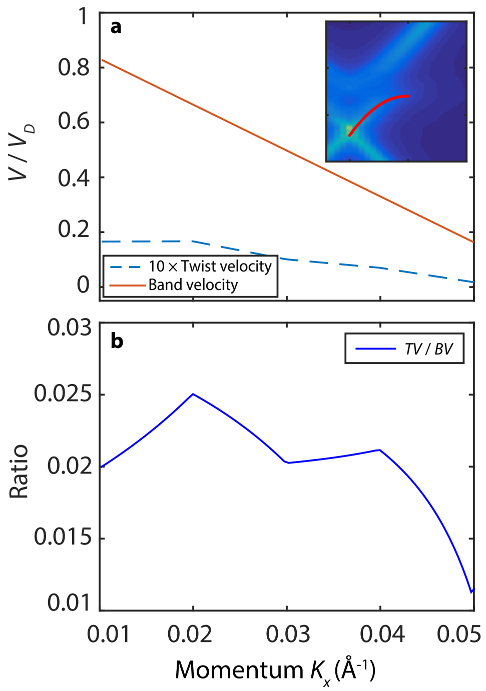

Supplementary Figure 3: **Twist velocity vs. band velocity**: **a**, Twist velocity and estimated band velocity of the upper D_0 Dirac cone from Fig. 3c of the main text ($U = -45$ eV, $\rho = 0.18\%$). **b**, The ratio of twist velocity to band velocity.

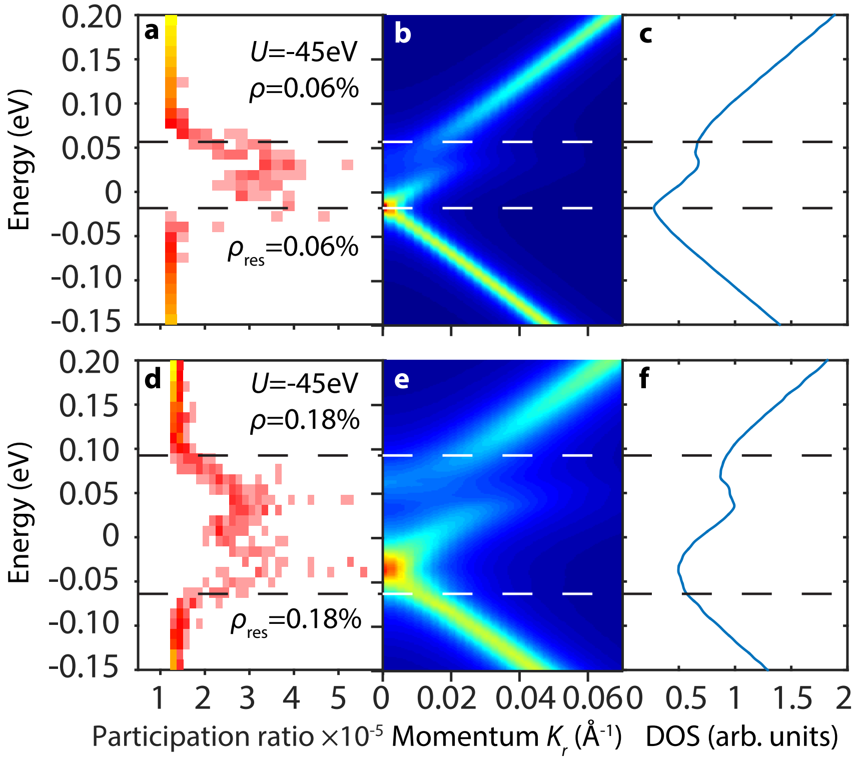

Supplementary Figure 4: **Defect density, DOS and band structure**: **a-c**, A histogram of energy-resolved participation ratio for a 350 × 350 site simulated surface with $\rho = 0.06\%$ density scalar defects is compared with the ARPES spectral function and DOS distribution. The number of states between the dashed lines is equal to twice the number of defects in the simulation. **d-f**, The same comparison is made for a $\rho = 0.18\%$ defect density.

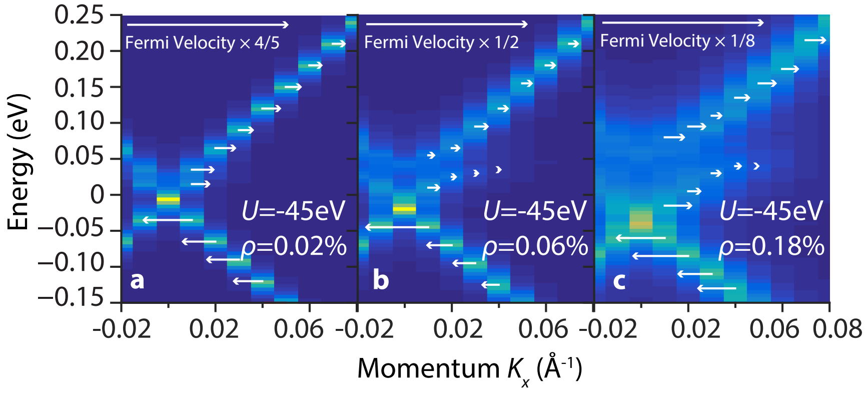

Supplementary Figure 5: **Band structure along the K_x axis**: **a-c**, The momentum resolved spectral function of a 300×300 site TI surface along the x-axis ($\mathbf{K} \parallel \mathbf{a}$) with randomly distributed point defects is shown as a function of defect density 'ρ'. Arrows indicate twist velocity $v_\theta(E, \mathbf{K})$.

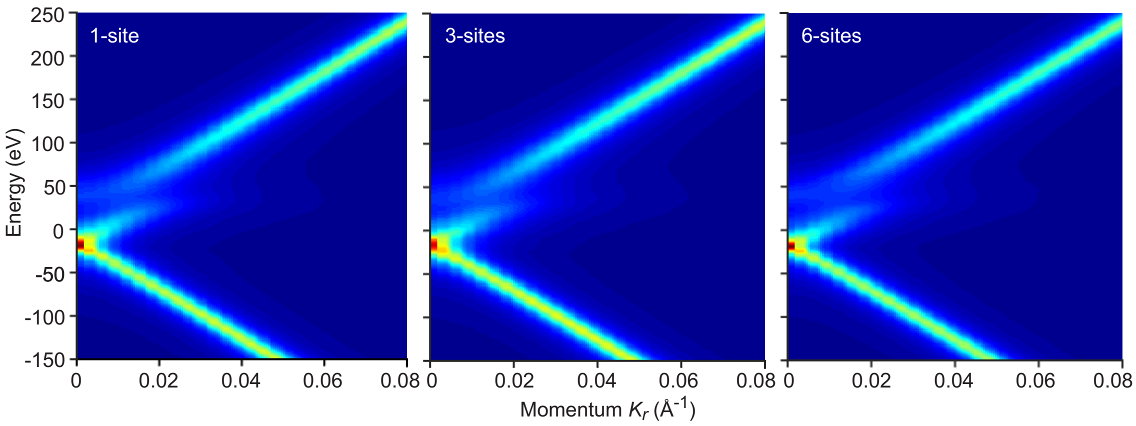

Supplementary Figure 6: The momentum space spectral function of a large TI surface with a $\rho = 0.06\%$ density of size (left) 1, (middle) 3, and (right) 6 defects. The defect potential is set to U=-55, -45, -40 eV, respectively, to maintain the same energy difference between the resonance state maximum and the Dirac point D_0.

SUPPLEMENTARY REFERENCES

[1] Alpichshev, Z. *et al.* STM Imaging of Impurity Resonances on Bi_2Se_3. *Phys. Rev. Lett.* **108**, 206402 (2012).

[2] Dai, J.-X. *et al.* Toward the Intrinsic Limit of the Topological Insulator Bi_2Se_3, *Phys. Rev. Lett.* **117**, 106401 (2016).

[3] Hsieh, D. *et al.*, A tunable topological insulator in the spin helical Dirac transport regime. *Nature* **460**, 1101-1105 (2009).

[4] Checkelsky, J. G., Ye, J., Onose, Y., Iwasa, Y., & Tokura, Y. Dirac-fermion-mediated ferromagnetism in a topological insulator. *Nat. Phys.* **8**, 729-733 (2012).

[5] Wray, L. A. *et al.* Observation of topological order in a superconducting doped topological insulator. *Nat. Phys.* **6**, 855859 (2010).

[6] Wray, L. A. *et al.*, A topological insulator surface under strong Coulomb, magnetic and disorder perturbations. *Nature Physics* **7**, 32-37 (2011).

[7] Fu, L. Hexagonal Warping Effects in the Surface States of the Topological Insulator Bi_2Te_3. *Phys. Rev. Lett.* **103**, 266801 (2009).

[8] Matsumoto, M., & Saito, M. A PRNG Specialized in Double Precision Floating Point Numbers Using an Affine Transition. Chapter in *Monte Carlo and Quasi-Monte Carlo Methods.* 589-602 (Springer Berlin Heidelberg, 2009), DOI:10.1007/978-3-642-04107-5_38.

[9] Anderson, E. *et al.*, LAPACK User's Guide (http://www.netlib.org/lapack/lug/lapack_lug.html), Third Edition, SIAM, Philadelphia, 1999.
